# Taxonomy, virulence determinants and antimicrobial susceptibility of *Aeromonas* spp. isolated from bacteremia in southeastern China

**DOI:** 10.1186/s13756-021-00911-0

**Published:** 2021-02-27

**Authors:** Yao Sun, Yajie Zhao, Wenya Xu, Renchi Fang, Qing Wu, Haokuang He, Chunquan Xu, Cui Zhou, Jianming Cao, Lijiang Chen, Tieli Zhou

**Affiliations:** 1 Department of Clinical Laboratory, the First Affiliated Hospital of Wenzhou Medical University, Wenzhou, Zhejiang Province China; 2 School of Laboratory Medicine and Life Sciences, Wenzhou Medical University, Wenzhou, Zhejiang Province China; 3 Department of Clinical Laboratory, The First Affiliated Hospital, Zhejiang University School of Medicine, Hangzhou, Zhejiang Province China

**Keywords:** *Aeromonas* spp., *Aeromonas dhakensis*, Bacteremia, Taxonomy

## Abstract

**Background:**

The study aimed to elucidate the species taxonomy, clinical manifestations, virulence gene profiles and antimicrobial susceptibilities of *Aeromonas* strains isolated from life-threatening bacteremia in southeastern China.

**Methods:**

Clinical samples of *Aeromonas* causing bacteremia were isolated from a teaching hospital in Wenzhou from 2013 to 2018 and a retrospective cohort study was performed. *Aeromonas* strains were identified at species level by housekeeping gene *gyrB*. Virulence and drug resistance-associated genes were screened by polymerase chain reaction (PCR) and antimicrobial susceptibility testing (AST) was performed by the VITEK 2 Compact system.

**Results:**

A total of 58 *Aeromonas* isolated from patients with bacteremia were collected during 6 years (2013–2018). 58 isolates were identified to five different species, where *Aeromonas dhakensis* appeared to be the predominant species (26/58), followed by *Aeromonas veronii* (13/58), *Aeromonas caviae* (10/58), *Aeromonas hydrophila* (7/58) and *Aeromonas jandaei* (2/58). 16 of 58 patients had poor prognosis. Poor prognosis was significantly associated with liver cirrhosis and inappropriate empirical antimicrobials therapy. The progression of bacteremia caused by *Aeromonas* was extremely fast, especially in *A. dhakensis* infections. Virulence genes *aer*, *lip*, *hlyA*, *alt*, *ast*, and *act*, were detected at ratios of 24.1% (14/58), 62.1% (36/58), 65.5% (38/58), 58.6% (34/58), 15.5% (9/58) and 65.5% (38/58), respectively. Antimicrobial susceptibility testing exhibited that 9 out of 58 isolates were identified as multi-drug resistant (MDR) organism. The *bla*_TEM_ gene was identified in all 9 MDR isolates. *bla*_SHV_, *bla*_AQU-1_, *bla*_MOX_, *bla*_CepH_, *bla*_CphA_ and *aac(6′)-Ib-cr* were detected in 4 isolates, 2 isolates, 1 isolate, 3 isolates, 8 isolates, and 3 isolates, respectively. The majority of *Aeromonas* strains maintained susceptible to 3rd generation cephalosporins, aminoglycosides, fluoroquinolones and furantoin.

**Conclusions:**

The prevalence and dangerousness of *Aeromonas* infections, especially *A. dhakensis*, are underestimated in clinic. Continuous monitoring is essential to keep track of MDR *Aeromonas* due to the increasing prevalence recently and a more effective measure is required to control the spread of resistance determinants.

## Backgroud

*Aeromonas* species are Gram-negative and rod-shaped bacteria, which are ubiquitous in aquatic environment, foodstuffs, and soil. *Aeromonas* are responsible for a variety of human infectious diseases, such as gastroenteritis, wound infections, hepatobiliary infections, necrotizing fasciitis and septicemia [1]. Humans carry *Aeromonas* species in their gastrointestinal tract. The carrying rate of *Aeromonas* in the feces of healthy people ranges from 0 to 4% [2]. Many infections caused by *Aeromonas* are self-limiting. However, in patients who have severe underlying diseases or immunocompromised individuals, invasiveness infections can be urgent and rapid-developing [3].

The *Aeromonas* taxonomy is complex. Nowadays, accurate laboratory identification is still a great challenge. Conventional biochemical tests, 16S rRNA sequencing and matrix-assisted laser desorption ionization-time of flight mass spectrometry (MALDI-TOF MS) analysis are unreliable in identifying *Aeromonans* at the species level. For example, *Aeromonas dhakensis* (formerly known as *Aeromonas aquariorum*) is often misidentified as *Aeromonas hydrophila* by traditional biochemical methods [4]. Accurate identification can be achieved by housekeeping genes sequencing, including *rpoD* and *gyrB*, or multilocus phylogenetic analysis (MLPA) [1].

Virulence factors produced by *Aeromonas* species are multifactorial, including adhesins, cytotoxins, hemolysins, lipases, proteases, the capacity to form biofilms, the use of specific metabolic pathways, and mediate virulence factor expression through quorum sensing [5]. The reported mortality rate among patients with *Aeromonas* bacteremia ranges from 24 to 63% [3]. *A. dhakensis* has been found prevalent in human infections and probably more lethal than other *Aeromonas* species in recent years. The pathogenicity of *Aeromonas* seems to be varied among different species levels. Moreover, along with the overuse of antimicrobials in agriculture, fish farming and clinical settings, increasing resistance has been noted in *Aeromonas* [6]. The antibiotic susceptibility varies with the geographical area and the species of *Aeromonas* tested [2]. Appropriate antimicrobials treatment is necessary to control the development of infections.

The prevalence and dangerousness of *Aeromonas* infections seems to be underestimated, as they vary among different geographic regions and types of infections [7], but fundamental reports are still insufficient in many countries. Wenzhou, a coastal city located in southeast China with subtropical climate, is prone to *Aeromonas* infection due to the humid subtropical climate. Incidences of bacteremia due to *Aeromonas* have been increasingly observed in Wenzhou with high morbidity and mortality in clinic. The present study aimed to investigate the clinical manifestations of bacteremia due to *Aeromonas* species over a 6-year period in a teaching hospital in southeastern China, and to assess the risk factors associated with mortality. Virulence gene determinants and antimicrobial susceptibility were also analyzed for the sake of advancing the understanding of *Aeromonas* causing bacteremia and establishing appropriate therapy strategy.

## Methods

### Bacterial strains and identification

This study was conducted at the First Affiliated Hospital of Wenzhou Medical University, a 4100-bed teaching hospital located in southeast China. A total of 58 isolates were obtained from patients with positive blood cultures for *Aeromonas* species between January 2013 and December 2018. The isolates were primarily identified using the MALDI-TOF MS (BioMérieux, Marcy I’ Etoile, France). Strains were further identified by housekeeping gene sequencing (*gyr*B). Strains used in this study were stored in 20% glycerol at − 80 °C.

### Data collection and definition

Retrospective cohort study was performed. The medical records of all patients with *Aeromonas* bacteremia were retrospectively reviewed and the following information was collected: demographics, symptoms and signs, monomicrobial or polymicrobial infection, antimicrobials susceptibility pattern and drugs application, source of infection, co-morbidities, and patient outcomes. Patient with the first positive blood culture collected within 48 h after admission were defined as community-acquired infection. Nosocomial infection was defined as the bacteremia episode detected at least 48 h after admission. Prognosis poor was defined as the death of a patient or the patient discharged from hospital due to continuously deteriorating conditions with a clinical course suggestive of persistently active infection. Prognosis well was defined as bacteremia associated symptoms improved without recurrence within 30 days [8, 9]. Antimicrobials treatments given before the antimicrobials susceptibility testing results became available were defined as empirical therapy. Inappropriate antimicrobials treatments were defined as the usage of those drugs which demonstrated ineffective against the causative isolates in vitro.

### Detection of genetic determinants related to virulence and drug resistance

The identified strains were recovered by streaking on nutrient agar plate and incubating for 24 h at 35 ℃. Total DNAs of *Aeromonas* isolated from bacteremia were obtained with an AxyPrep Bacterial Genomic DNA Miniprep kit (Axygen Scientific, Union City, CA, USA) and were used as polymerase chain reaction (PCR) templates for subsequent gene detection. Six virulence associated genes were selected as virulence markers, including aerolysin (*aerA*), heat-stable cytotonic enterotoxin (*ast*), heat-labile cytotonic enterotoxin (*alt*), cytotoxic enterotoxin (*act*), hemolysin (*hlyA*), and phospholipase (*lip*). The presence of the β-lactamase genes (*bla*_TEM_, *bla*_SHV_, *bla*_CTX-M-1_, *bla*_AQU-1_, *bla*_MOX_, *bla*_CepH_, *bla*_IMP_, *bla*_VIM_ and *bla*_CphA_), and plasmid-mediated quinolone resistance genes (*qnrA*, *qnrB*, *qnrD* and *aac(6′)-Ib-cr*) was also analyzed. Primer sequences for the amplification were as previously described [8, 10–12]. The positive PCR amplicons were sequenced by Shanghai MajorbioBioPharm Technology Co. (Shanghai, China). The sequences were blasted using BLAST at NCBI website (http://blast.ncbi.nlm.nih.gov/Blast.cgi).

### Antimicrobial susceptibility testing (AST)

The antimicrobial susceptibility patterns of all isolates to a panel of antimicrobials were determined using the VITEK 2 Compact System, including ampicillin (AMP), ampicillin/sulbactam (SAM), ceftriaxone (CRO), ceftazidime (CAZ), cefotetan (CTT), cefazolin (CZO), cefepime (FEP), piperacillin/tazobactam (TZP), aztreonam (ATM), imipenem (IPM), levofloxacin (LEV), ciprofloxacin (CIP), Trimethoprim/sulfamethoxazole (SXT), amikacin (AMK), gentamicin (GEN), tobramycin (TOB) and furantoin (NIT). The breakpoints were interpreted according to the Clinical and Laboratory Standards Institute (CLSI) 2018 guidelines.

### Phylogenetic and statistical analysis

The positive PCR amplicons (*gyrB*, virulence determinants, and drug resistance genes) were sequenced by Shanghai MajorbioBioPharm Technology Co. (Shanghai, China). Nucleotide sequences were analyzed and compared using BLAST (http://www.ncbi.nlm.nih.gov/BLAST). A phylogenetic tree was generated using the unrooted neighbor-joining method with the Kimura's 2-parameter method by Mega 5.0 software. Bootstrap values were calculated by 1000 replicates [13]. Statistical analyses were performed using SPSS, version 17.0 (SPSS Inc., Chicago, IL, USA). Pearson’s Chi-square test was used to examine categorical variables and Student’s t test or Mann–Whitney U test was used for continuous variables. Variables with statistical significance in univariate analysis were submitted to multivariate analysis. Risk factors for prognosis of *Aeromonas* bacteremia were analyzed with multivariate logistic regression models. Odds ratios (OR) were calculated with 95% confidence interval. A *P* value of < 0.05 was regarded as statistically significant.

## Results

### *Aeromonas* diversity

**Fig. 1:**
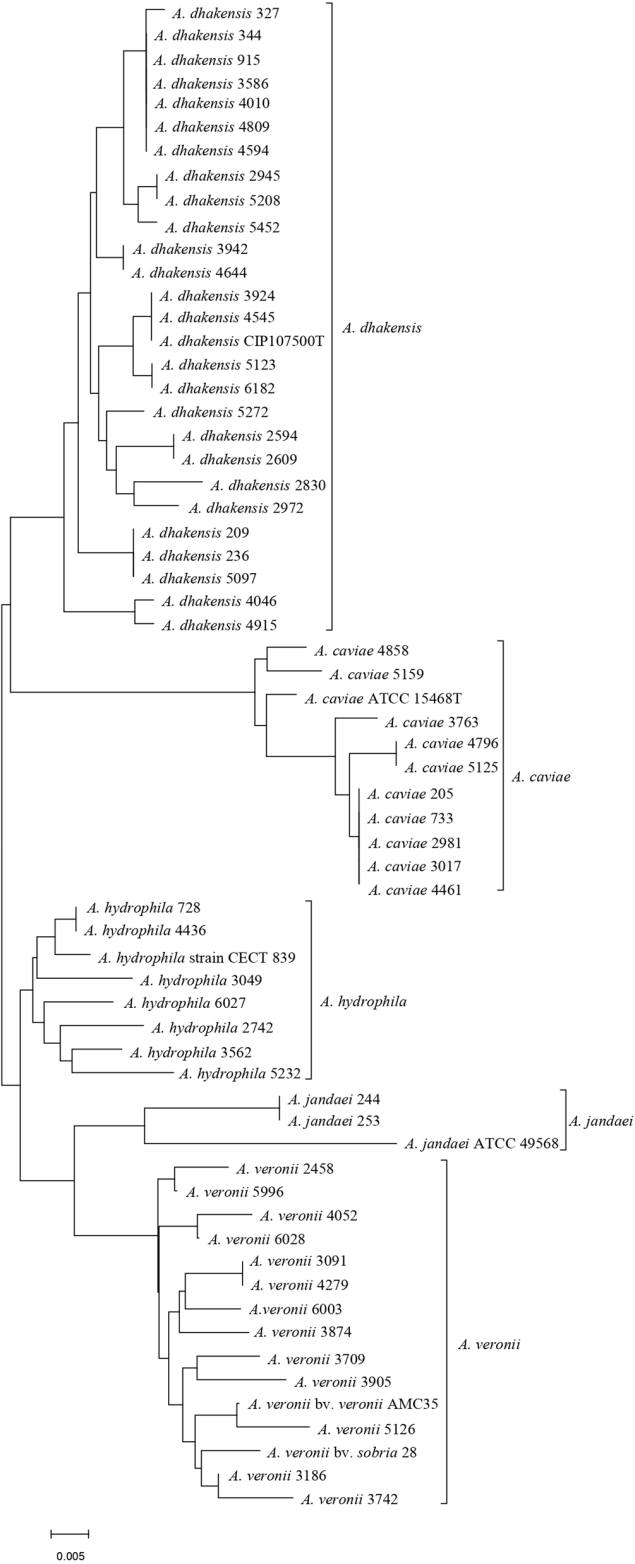
Phylogenetic tree based on *gyrB* gene sequences. Phylogenetic tree was constructed by the neighbor-joining method with Kimura's 2-parameter method. Scale bar represents 0.05 substitutions per site. Bootstrap values above 50% are shown (n = 1000 bootstrap replicates)

Phylogenetic tree based on housekeeping gene *gyrB* exhibited that all 58 isolates were divided into 5 different species, with the predominant species being *A. dhakensis* (26/58). Besides, 13 isolates of *Aeromona*s *veronii*, 10 isolates of *Aeromonas caviae*, 7 isolates of *A. hydrophila* and 2 isolates of *Aeromonas jandaei* were identified at the species level (Fig. 1). The MALDI-TOF MS system showed poor coincidence with housekeeping gene sequencing analysis at the species level. The concordance rate between MALDI-TOF and *gyrB* sequencing was 53.4%. *A. dhakensis* was incorrectly identified as *A. hydrophila* by MALDI-TOF MS. Moreover, two *A. jandaei* strains were misidentified as *A. hydrophila* or *Aeromonas veronii*.

### Characteristics of investigated patients

During the investigated period, 58 patients were detected with positive blood culture of *Aeromonas*. 16 patients had poor prognosis (death or therapy failure), where *A. dhakensis* (12/26) was the most common *Aeronomas* species, followed by *A. veronii* (2/13), *A. caviae* (1/10), *A. hydrophila* (1/7) and *A. jandaei* (0/2). The average age of the 58 patients with positive blood culture was 61 ± 16.7 years old and the percentage of male patients was 70% (40/58). Polymicrobial infections were detected in nine cases, which were co-infected with *Klebsiella pneumoniae* (3 cases), *Escherichia coli* (3 cases). *Proteus vulgaris* (1 case), *Klebsiella oxytoca* (1 case) and *Enterobacter cloacae* (1 case). Univariate analysis indicated that significant differences were observed within the parameters of liver cirrhosis, inappropriate empirical antimicrobials treatment, thrombocytopenia, the community-acquired infection, and clinical outcomes (Septic shock, admission to ICU and the length of stay in hospital). Multivariate logistic regression analysis showed that poor prognosis was only significantly associated with liver cirrhosis (OR = 7.41, 95% CI, 1.32–41.55, *P* < 0.05) and inappropriate empirical antimicrobials (OR = 16.91, 95% CI, 3.04–94.22, *P* < 0.05). The exact characteristics of patients were listed in Table 1. The outcomes of *A. dhakensis* bacteremia were worsen than other species (*P* < 0.05).

**Table 1:** Clinical characteristics and risk factors of 58 patients with bacteremia caused by *Aeromonas* species

Clinical characteristic	No (%) of all patients (n = 58)	Prognosis		*P-*value	Multivariate analysis		*P*-value
		Poor (n = 16)	Well (n = 42)		Odds ratios	95% CI	
Gender							
Male	40 (70.0)	12 (75.0)	28 (66.7)	0.752	–	–	–
Female	18 (30.0)	4 (25.0)	14 (33.3)				
Age, years (means ± SD)	61.1 ± 16.7	56.56 ± 14.45	62.76 ± 17.30	0.208	–	–	–
Age							
< 65	30 (51.7)	10 (62.5)	20 (47.6)	0.311	–	–	–
≥ 65	28 (48.3)	6 (37.5)	22 (52.4)				
Symptoms and signs							
Fever (> 39℃)	31	6 (37.5)	25 (59.5)	0.133	–	–	–
Leukocytosis	26	6 (37.5)	20 (47.6)	0.489	–	–	–
Thrombocytopenia	34	13 (81.3)	21 (50)	0.031*			
Neutropenia	19	7 (43.8)	12 (28.6)	0.271	–	–	–
Microbial findings							
Monomicrobial	49 (84.5)	16 (100.0)	33 (78.6)	0.051	–	–	–
Polymicrobial	9 (15.5)	0 (0.0)	9 (21.4)				
Antimicrobial susceptibility							
MDR	9 (15.5)	4 (25.0)	5 (11.9)	0.243	–	–	–
Non MDR	49 (84.5)	12 (75.0)	37 (88.1)				
Source of infection							
Community acquired	34 (58.6)	13 (81.3)	21 (50)	0.031*	3.201	0.57–18.00	0.187
Nosocomial infection	24 (41.4)	3 (18.8)	21 (50)				
Co-morbidity							
Liver cirrhosis	26 (44.8)	11 (68.8)	15 (35.7)	0.024*	7.41	1.32–41.55	0.023*
Diabetes mellitus	7 (12.1)	2 (12.5)	5 (11.9)	1.000	–	–	–
Malignancy	18 (31.0)	6 (37.5)	12 (28.6)	0.538	–	–	–
Leukemia	8 (13.8)	3 (18.8)	5 (1.9)	0.672	–	–	–
Treatment							
inappropriate empirical antimicrobials	15 (25.9)	10 (62.5)	5 (11.9)	0.000*	16.91	3.04–94.22	0.001*
Clinical outcomes							
Septic shock	14	11 (68.8)	3 (7.1)	0.000*	–	–	–
Admission to ICU	10	9 (90)	1 (2.4)	0.000*	–	–	–
Length of stay in hospital, days	17 (6–24.75)	2.5 (1–6)	19 (11.25–30)	0.000*	–	–	–

Values are presented as No. (%), mean ± SD or median (25th–75th percentile) of patients. * significant

### Distribution of virulence and drug resistance determinants

**Fig. 2:**
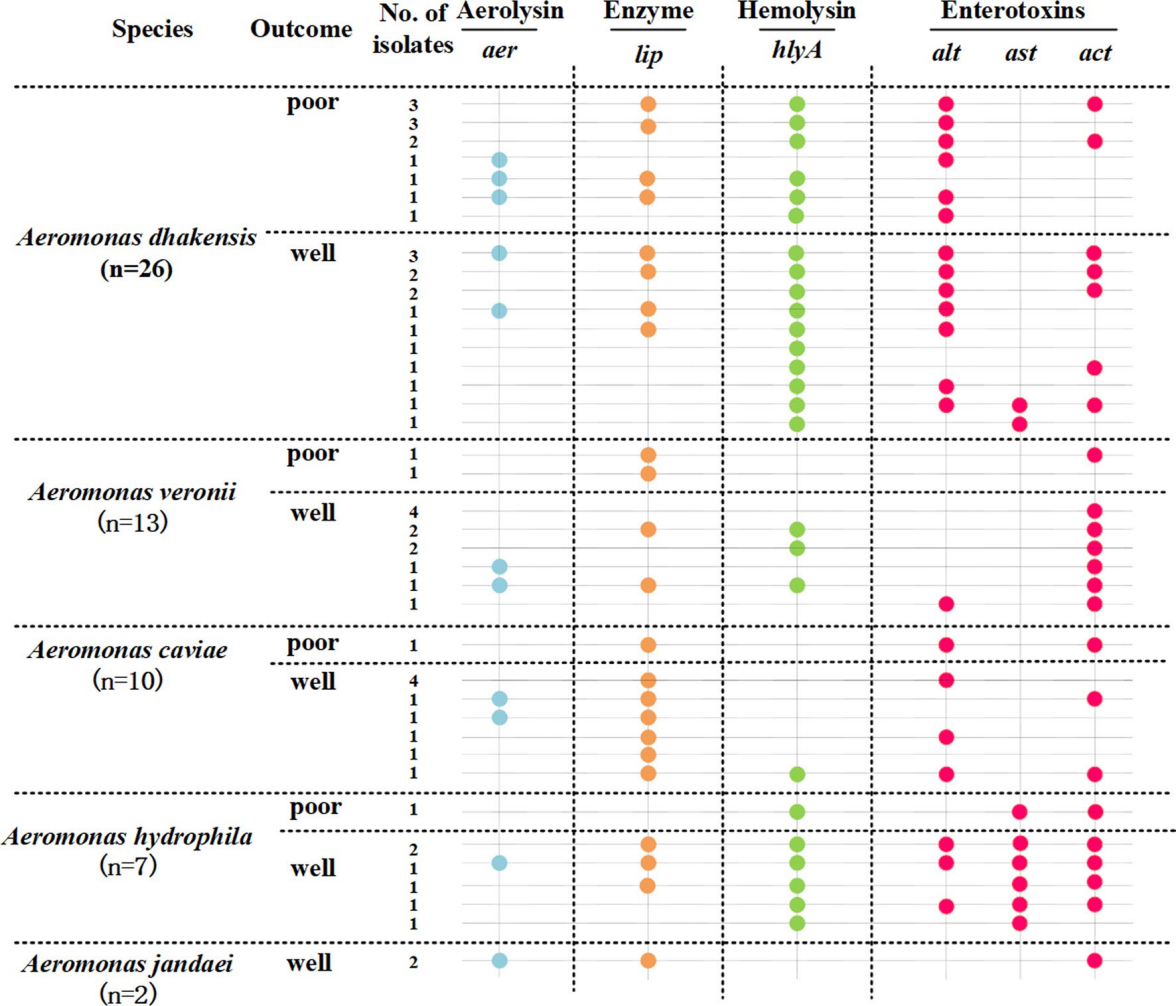
Virulence genes profile of 58 Aeromonas isolates. Virulence genes identified by PCR-based profiling are shown by colored dots

Virulence encoding genes, including *aer*, *lip*, *hlyA*, *alt*, *ast*, and *act*, were detected at ratios of 24.1% (14/58), 62.1% (36/58), 65.5% (38/58), 58.6% (34/58), 15.5% (9/58) and 65.5% (38/58), respectively. Virulence genes profile of 58 *Aeromonas* isolates was showed in Fig. 2. At least one virulence determinants was found in all 58 isolates. The gene *hlyA* and *act* were most prevalent in these isolates. Single virulence gene was detected in 12.1% (7/58) of isolates, and more than two virulence genes were found in remaining strains. There was no significant difference in virulence genes between strains isolated from patients with poor prognosis and those with well prognosis. Additionally, no statistical significance was observed in the prevalence of all the studied virulence genes between isolates separated from community acquired and nosocomial infection. We found 27 different combination patterns (PTs) of six examined genes. The two most prevalent PT (n ≥ 5) were PT1 *(lip*/*hlyA*/*alt*/*act*, n = 6) and PT2 (*lip*/*alt*, n = 5). Only one isolate of *A. hydrophila* carried all the investigated virulence genes, and the patient was cured after 32 days of hospitalization. Notably, 5 of 6 isolates grouped into PT1 were *A. dhakensis*, among which 3 lead to poor prognosis. The *bla*_TEM_ gene was identified in all 9 MDR isolates. *bla*_SHV_, *bla*_AQU-1_, *bla*_MOX_, *bla*_CepH_, *bla*_CphA_ and *aac(6′)-Ib-cr* were detected in 4 isolates, 2 isolates, 1 isolate, 3 isolates, 8 isolates, and 3 isolates, respectively.

### Antimicrobial susceptibility profiles

Antimicrobials susceptibility testing exhibited that the majority of the 58 isolates maintained susceptible to aminoglycosides, fluoroquinolones and furantoin (Table 2). Resistance to ceftazidime, cefotetan, ceftriaxone, cefepime, piperacillin/tazobactam, aztreonam were 10.3%, 13.8%, 15.5%, 1.7%, 10.3% and 5.2%, respectively. No significant increase in resistance during six years was observed. 9 out of 58 isolates were identified as multi-drug resistant (MDR) organism, including 4 isolates of *A. dhakensis*, 3 *A. hydrophila*, 1 *A. veronii*, and 1 *A. caviae*. Among which, six MDR strains were isolated in 2017 and 2018. The first MDR strain was recovered from a 78-year-old woman with community-acquired infection in 2013. 24.1% (14/58) isolates were non-susceptible to imipenem.

**Table 2:** Antimicrobial susceptibility patterns of 58 Aeromonads separated from bacteremia

Antimicrobial agent	CLSI breakpoint interpretation (%)			MIC50	MIC range
	S	I	R		
Ceftazidime	88	1.7	10.3	≤ 1	≤ 1 ~ ≥ 64
Cefotetan	86.2	0	13.8	≤ 4	≤ 4 ~ ≥ 64
Ceftriaxone	79.3	5.2	15.5	≤ 1	≤ 1 ~ ≥ 64
Cefepime	98.3	0	1.7	≤ 1	≤ 1 ~ 32
Piperacillin/tazobactam	88	1.7	10.3	≤ 4	≤ 4 ~ ≥ 128
Aztreonam	91.4	3.4	5.2	≤ 1	≤ 1 ~ ≥ 64
Imipenem	75.9	10.3	13.8	≤ 1	≤ 1 ~ ≥ 16
Levofloxacin	96.6	1.7	1.7	≤ 0.25	≤ 0.25 ~ ≥ 8
Ciprofloxacin	96.6	0	3.4	≤ 0.25	≤ 0.25 ~ ≥ 4
TRIMETHOPRIM/sulfamethoxazole	87.9	0	12.1	≤ 20	≤ 20 ~ ≥ 320
Amikacin	100	0	0	≤ 2	≤ 2
Gentamicin	100	0	0	≤ 1	≤ 1
Tobramycin	93.1	5.2	1.7	≤ 1	≤ 1 ~ ≥ 16
Furantoin	100	0	0	≤ 16	≤ 16

## Discussion

*Aeromonas* spp. are of increasing importance for causing multiple of clinical infections, including diarrhea, soft tissue infection, and bacteremia. *Aeromonas* bacteremia is an urgent, rapid-developing disease with high mortality [14]. Moreover, according to similar clinical manifestations, *Aeromonas* infections are often misdiagnosed as Vibrio infections before microbiology identification by laboratory, which may lead to improperly use of antimicrobials and ineffective treatment [14]. In this study, four patients were misdiagnosed as *Vibrio vulnificus* infections before laboratory identification. The symptoms progressed rapidly and these patients were severely inflamed with ecchymosis and blisters in 2 days. Unfortunately, all of them developed into multiple organ dysfunction syndrome (MODS) and resulted in poor prognosis. Coincidentally, they all got infected in community and suffered from liver cirrhosis. Among whom, one patient received ineffective empirical therapy by using imipenem alone. This pathogenic *Aeromonas* isolate was subsequently confirmed to produce *Aeromonas* spp. specific “Carbapenem hydrolyzing *Aeromonas*” metallo-beta-lactamase (CphA) [15] and to be resistant to imipenem in vitro while remaining susceptible to many other antimicrobials, such as the third- cephalosporins, quinolones and aminoglycosides. *Aeromonas* infections are reported to be prevalent in regions with a high prevalence of chronic hepatitis and warm climate, like Taiwan, which is regarded as one of the endemic areas [16]. However, in mainland China, the incidence of *Aeromonas* bacteremia in human beings remains to be elucidated. Wenzhou is in the southeastern coastal area with subtropical climate. Increasing prevalence of *Aeromonas* bacteremia has been found with high morbidity and mortality in the hospital studied.

*Aeromonas* are not difficult to isolate, but identification at species level is challenging due to its phenotypic heterogeneity. Compared with the use of 16 s rRNA gene, nucleotide sequencing of housekeeping genes, such as *gyrB*, *rpoB* and *rpoD*, can provided a more definitive identification of the genus [17]. Several researches have shown that MALDI-TOF MS could efficiently identify *A. dhakensis*, which is often clinically misidentified as *A. hydrophila* by phenotypic methods [4]. Nevertheless, *A. dhakensis* couldn’t be identified by MALDI-TOF MS in this study, possibly because it hasn’t been included in the commercial database of BioMérienx system. Housekeeping gene *gyrB* sequencing exhibited that *A. dhakensis* was the most common *Aeromonas* species, followed by *A. veronii*. This is in contrast to the previous reports in which the authors stated that *A. hydrophila* and *A. caviae* were the most frequent *Aeromonas* species causing bacteremia in Taiwan, and *A. caviae* was the most common pathogen contributing to *Aeromonas* bacteremia in Japan [18]. Notably, *A. dhakensis* and *A. jandaei* were misidentified as *A. hydrophila* or *A. veronii* by MALDI-TOF MS. The patients with bacteremia caused by *A. dhakensis* are reported to have a higher sepsis-related mortality rate than those with other species in recent years, with the application of molecular biological method [19]. Similarly, bacteremia caused by *A. dhakensis* is more lethal than other species in our research. Notably, the importance of *A. dhakensis* in human infections might be seriously underrated and should be re-evaluated along with the changing taxonomy, and more accurate epidemiological researches are needed to establish the bacteriology distribution of *Aeromonas* bacteremia in different regions.

In our retrospective analysis, the average age of the 58 patients with positive blood culture was 61.1 ± 16.7 years old, suggesting that older people were more susceptible than younger individuals. However, no significant difference was found in age between prognosis the poor group and the prognosis well group (P > 0.05). 40 out of 58 patients were male, which may attribute to that alcoholic cirrhosis was more prevalent in male than female in our study. Similar to previous researches [20], we also found that the majority of patients had a variety of underlying diseases, including liver cirrhosis, diabetes mellitus, under immunosuppressed conditions, leukemia and other kinds of malignancy. Nearly half of patients in this study were diagnosed with liver cirrhosis. In accordance with previous research [14], our study exhibited that *Aeromonas* bacteremia accounted for significant morbidity and mortality in cirrhotic patients, suggesting that patients with liver cirrhosis are at risk of developing *Aeromonas* bacteremia. Moreover, initial inappropriate empirical antimicrobial usage was associated with poor outcomes for patients with *Aeromonas* bacteremia. The prevalence and high mortality rate of *Aeromonas* bloodstream infections in cirrhotic patients might be a consequence of dysregulated intestinal bacterial translocation and cirrhosis associated immune dysfunction (CAID) [21]. Among the 58 patients with *Aeromonas* bacteremia in this study, four patients were claimed to be dead in hospital, and 12 had dismal prognosis and then discharged without treatment. Polymicrobial infection didn’t result in worse prognosis than monomicrobial infection (*P* > 0.05). We found that consumption of sea food, trauma exposed or contact with water contaminated with *Aeromonas* [15], preexisting liver cirrhosis were the potential risk factors of *Aeromonas* infections or even lead to more rapid infection progresses. Additionally, length of hospital stays of community-acquired infections with poor prognosis ranged from 1 to 7 days (median 2 days), indicating that community-acquired infections developed more rapidly and lethally. No statistical significance in prognosis was observed between MDR and non-MDR strains. Compared to antimicrobial susceptibility, the pathogenicity of pathogens and the health status of the patients were probably more critical to the prognosis.

The pathogenicity of *Aeromonas* is multi-factorial, complex and may be associated with different interaction of various virulence factors acting either synergy or alone. The majority of *Aeromonas* isolates investigated in this study possess more than two virulence genes and seven strains harbor only one single gene. Isolates carrying more virulence genes didn’t mean higher pathogenicity. One patient died of an *A. veronii* strain, which only possess lipase encoding gene *lip*, six days after admission to ICU. However, another one infected by *A. hydrophila* carrying all the studied virulence determinants was cured after 32 days of hospitalization. The most obvious difference between these two patients was that the former suffered from liver cirrhosis. However, it may be explained by different expression level of the genes or interaction with other virulence factors not included in this study. Inconsistent with the previous study [7], no particular pattern of virulence genes was observed in this study.

Except for ceftriaxone (79.3%) and imipenem (75.9%), more than 80% of the isolates were susceptible to all remaining antimicrobials studied. In spite of intrinsical resistance to many antimicrobials, *Aeromonas* maintained well susceptible to most antimicrobials generally used in clinic. Relatively high carbapenem resistance rate may be due to the carriage of *cphA* [15]. Considering the rapid symptom progression and *Aeromonas* spp. specific drug resistance mechanisms, it is important to select the most appropriate antimicrobials usage or surgical intervention to prevent or cure *Aeromonas* infections as soon as possible, especially in patients with liver cirrhosis. Antimicrobial susceptibility patterns of *Aeromonas* spp. exhibited best susceptibility to aminoglycosides, suggesting that aminoglycosides might be recommended for empirical therapy of *Aeromonas*-associated bacteremia. 6 out of 9 MDR strains were isolated in 2017 and 2018, While the trends of antimicrobial susceptibilities among the years were stable without significant changes. Moreover, the resistance of bacteria associated with food animals and environments to antimicrobial agents represents a potential health threat [22]. It raises an alert for the developing of multidrug resistant strains in *Aeromonas* spp. isolated from clinic.

## Conclusions

Considering the high morbidity and mortality, people should attach great importance to bacteremia caused by *Aeromonas* spp., especially in those immunocompromised patients with severe underlying diseases. Identification of *Aeromonas* at the species level is important for predicting clinical severity and outcome. The increasing emergence of MDR strains in recent years requires more attention and monitoring.

## Data Availability

All data generated or analyzed during this study are included in this published article.

## Abbreviations

AMK: Amikacin
AMP: Ampicillin
AST: Antimicrobial Susceptibility Testing
ATM: Aztreonam
CAID: Cirrhosis Associated Immune Dysfunction
CAZ: Ceftazidime
CLSI: The Clinical and Laboratory Standards Institute
CIP: Ciprofloxacin
CRO: Ceftriaxone
CTT: Cefotetan
CZO: Cefazolin
FEP: Cefepime
GEN: Gentamicin
ICU: Intensive Care Unit
IPM: Imipenem
LEV: Levofloxacin
MALDI-TOF MS: Matrix-Assisted Laser Desorption Ionization-Time of Flight Mass Spectrometry
MDR: Multi-Drug Resistant
MLPA: Multilocus Phylogenetic Analysis
NIT: Furantoin
OR: Odds ratios
PCR: Polymerase Chain Reaction
SAM: Ampicillin/Sulbactam
SXT: Trimethoprim/sulfamethoxazole
TOB: Tobramycin
TZP: Piperacillin/Tazobactam
